# Supplementary material for: Insights into Non-Proteolytic Inhibitory Mechanisms of Polymorphic Early-Stage Amyloid β Oligomers by Insulin Degrading Enzyme
Source: Biomolecules. 2022 Dec 16;12(12):1886. doi: 10.3390/biom12121886 (PMC9776231; doi:10.3390/biom12121886)
Supplement: Supplementary file 1 [file biomolecules-12-01886-s001.zip › biomolecules-2078206-supplementary.pdf]

Supporting Information for

"Insights into non-proteolytic inhibitory mechanisms of polymorphic early-stage amyloid β oligomers by insulin degrading enzyme"

Karina Abramov-Harpaz[1,2,3] Yifat Miller[1,2,3*]

[1]Department of Chemistry, Ben-Gurion University of the Negev, P.O. Box 653, Be'er Sheva 84105, Israel

[2] The School of Brain Sciences and Cognition, Ben-Gurion University of the Negev, Beér-Sheva 84105, Israel

[3] Ilse Katz Institute for Nanoscale Science and Technology, Ben-Gurion University of the Negev, Beér-Sheva 84105, Israel

Corresponding author:

*Yifat Miller: ymiller@bgu.ac.il

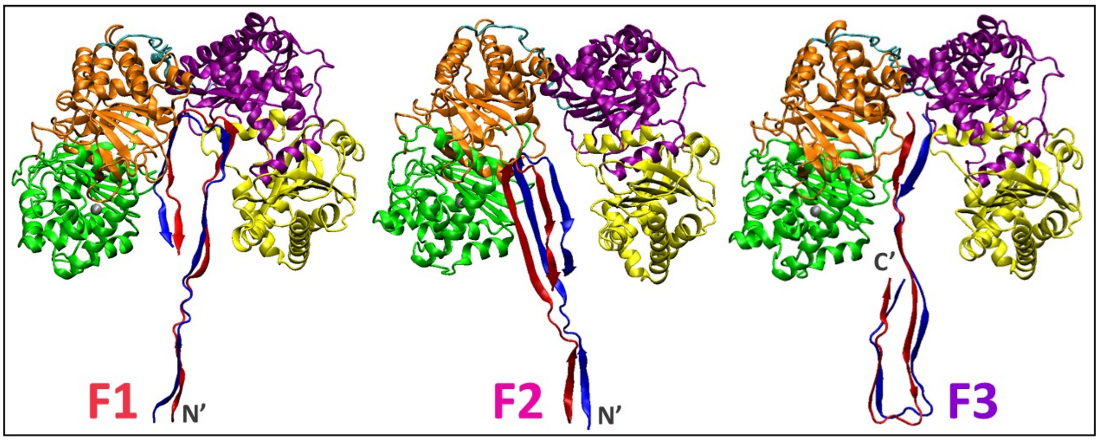

Figure S1: Three initial minimized structures of IDE-Aβ fibril-like dimer complexes that differ by the insertion of Aβ fibril-like dimer in 3 different orientations. In model F1 – the C-termini domains of Aβ dimer were inserted towards the IDE-C domain. In model F2 – the C-termini domains of Aβ dimer were inserted towards IDE-N domain. In model F3 – the N-termini domain of Aβ dimer were inserted both to IDE-N and IDE-C domains.

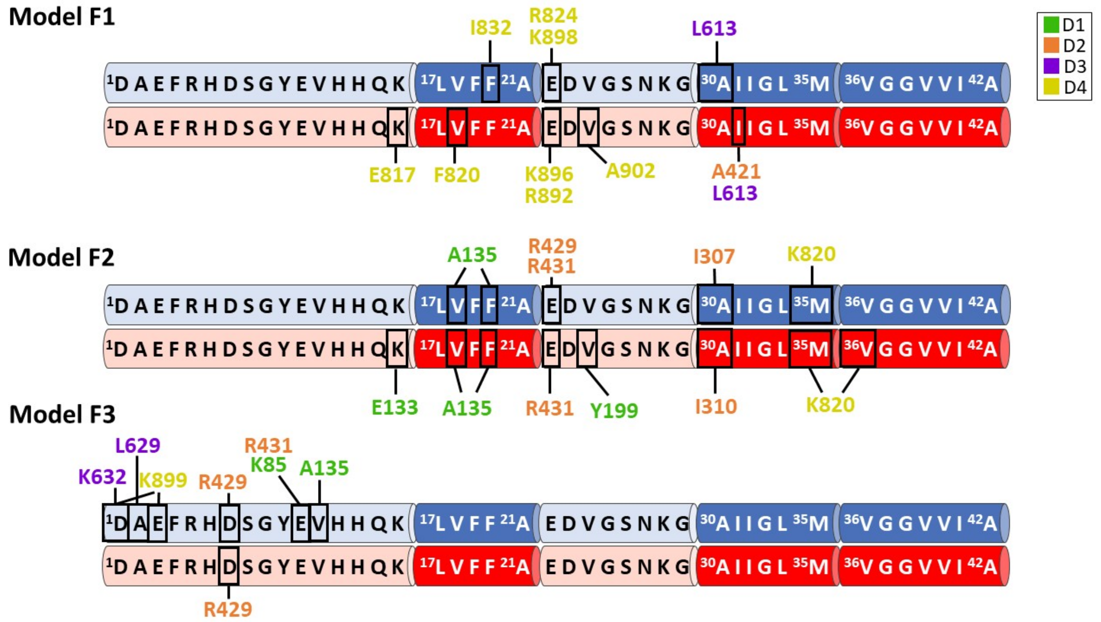

Figure S2: The residues of IDE within the four domains (domain 1: green; domain 2: orange; domain 3: purple; domain 4: yellow) that bind residues in Aβ fibril-like dimer in three models: F1, F2 and F3.

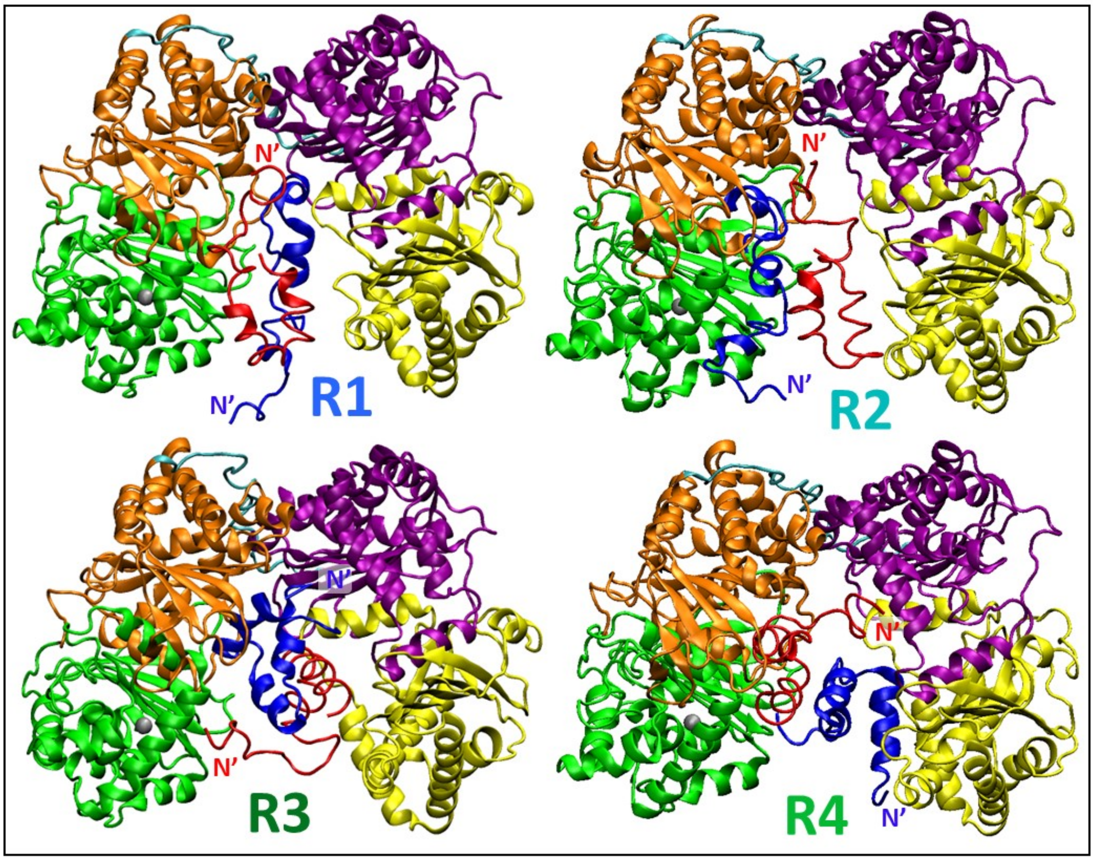

Figure S3: Four initial minimized structures of IDE-Aβ random coil/α-helix dimer complexes. In models R1 and R2, the random coil/α-helix dimer (model D1) was inserted into the IDE in two different orientations. In models R3 and R4, the random coil/α-helix dimer (model D4) was inserted into the IDE in two different orientations. Models D1 and D4 of the random coil/α-helix Aβ dimer were taken from: Press-Sandler, O.; Miller, Y., Distinct Primary Nucleation of Polymorphic Aβ Dimers Yields to Distinguished Fibrillation Pathways. *ACS Chem. Neurosci.* 2019, 10, 4407-4413.

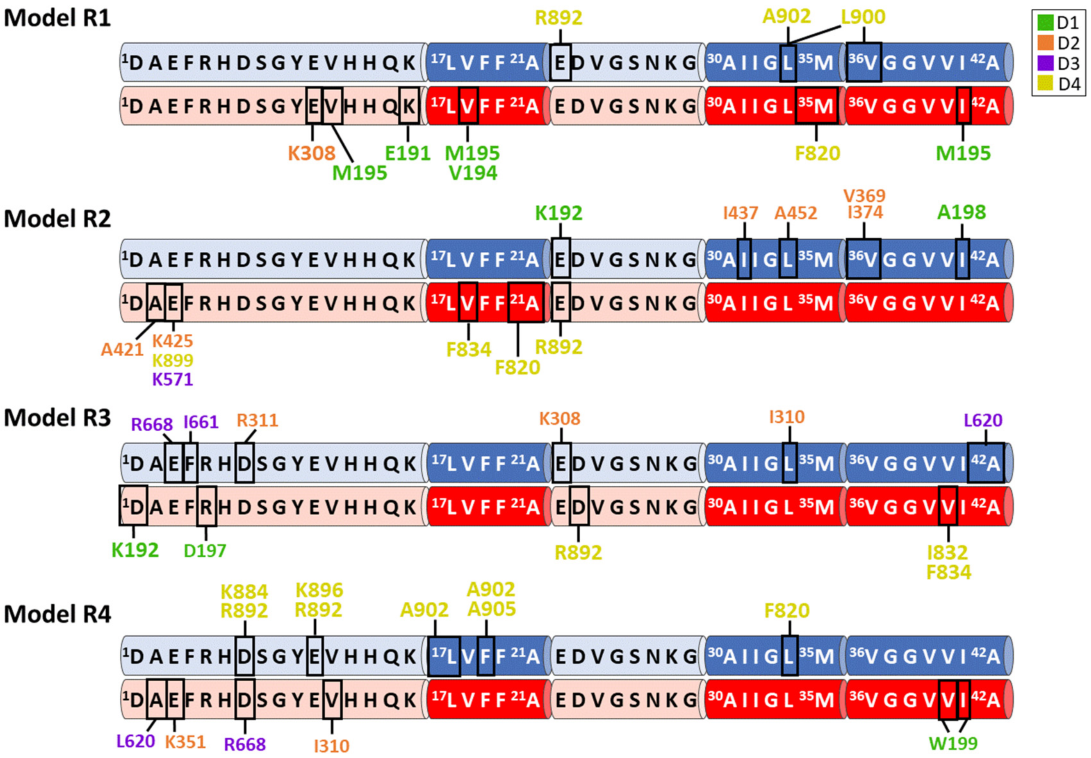

Figure S4: The residues of IDE within the four domains (domain 1: green; domain 2: orange; domain 3: purple; domain 4: yellow) that bind residues in Aβ random coil/α-helix dimer in four models: R1, R2, R3, and R4.

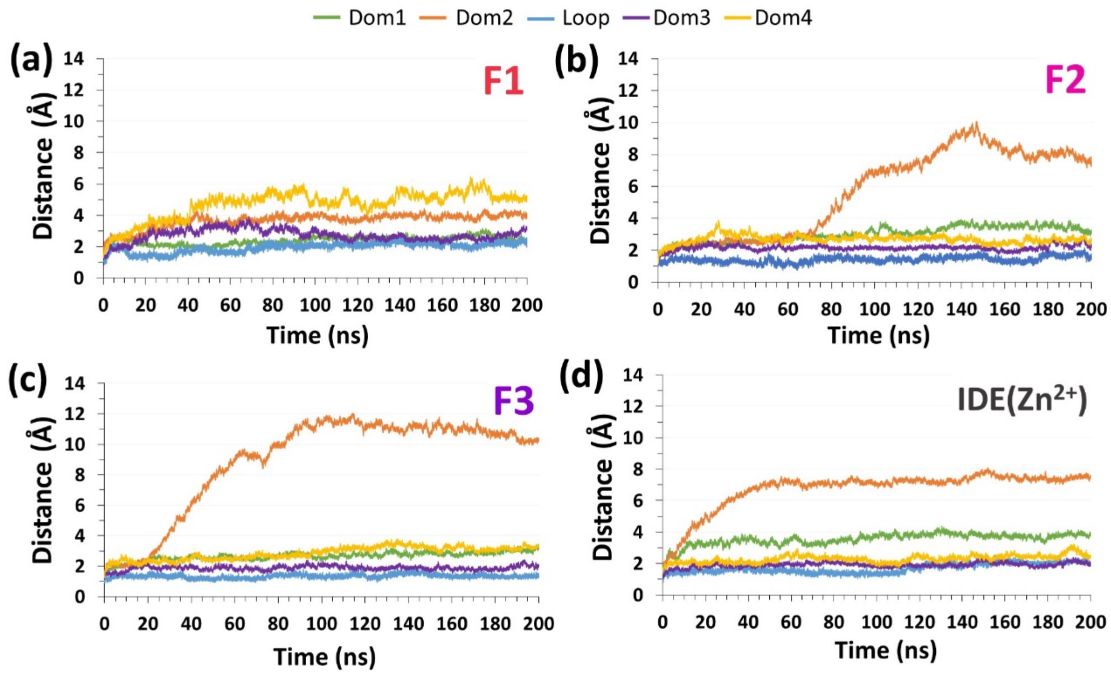

Figure S5: The root-mean-square deviations (RMSDs) values of IDE domains along the MD simulations for the models (a) F1 (b) F2 (c) F3, and (d) IDE in absence of Aβ dimer: domain 1 (color: green), domain 2 (color: orange), loop (color: cyan), domain 3 (color: purple), domain 4 (color: yellow).

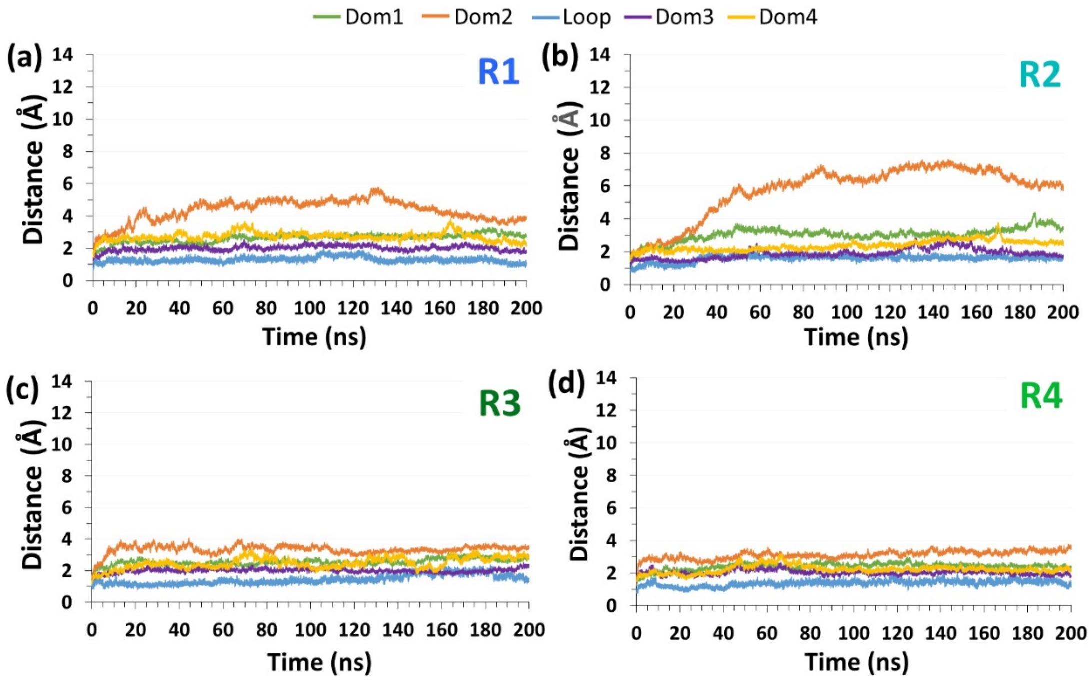

Figure S6: The root-mean-square deviations (RMSDs) values of IDE domains along the MD simulations for the models (a) R1, (b) R2, (c) R3, and (d) R4: domain 1 (color: green), domain 2 (color: orange), loop (color: cyan), domain 3 (color: purple), domain 4 (color: yellow).

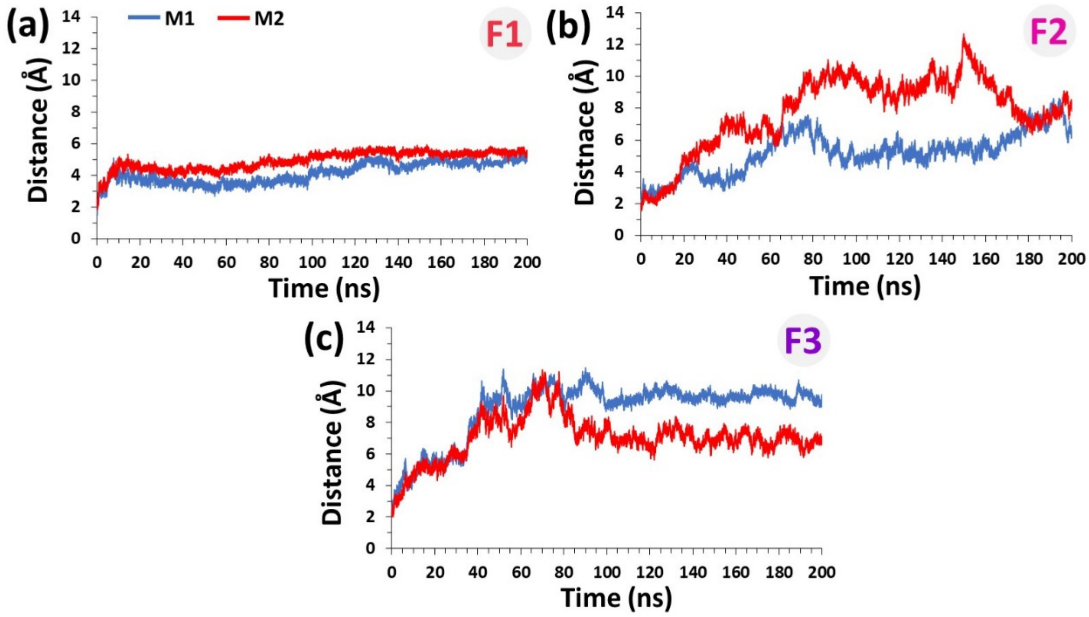

Figure S7: The root-mean-square deviations (RMSDs) values for Aβ dimers in models: (a) F1, (b) F2, and (c) F3, along the MD simulations.

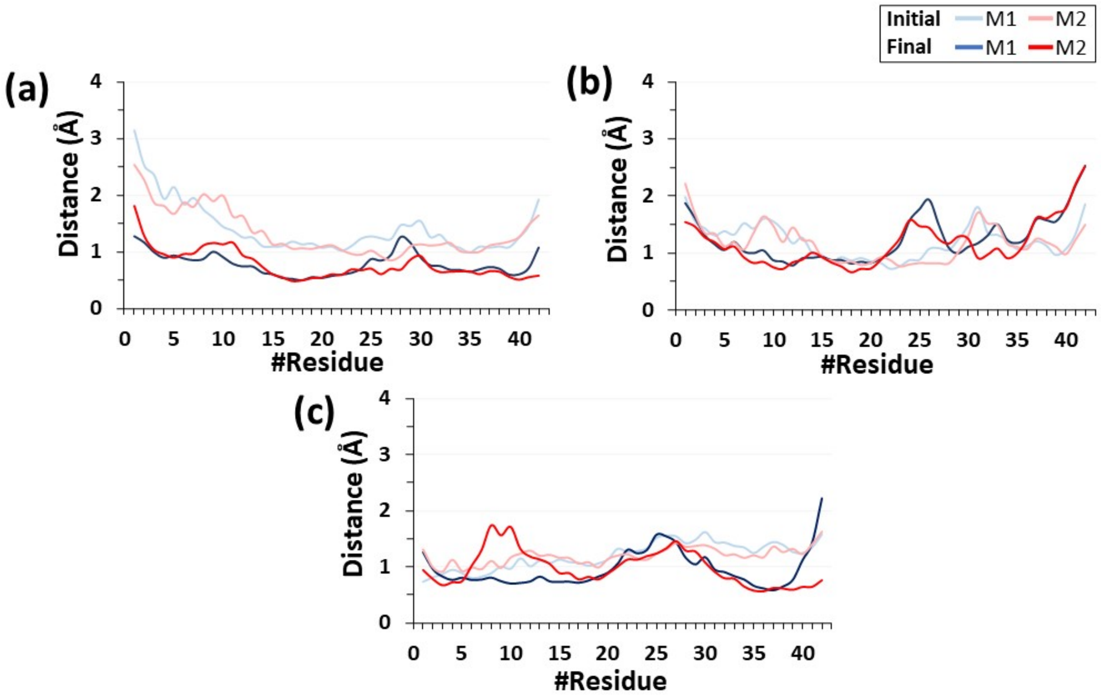

Figure S8: Root-mean-square fluctuations (RMSFs) of Aβ monomers M1 and M2 within each dimer, in the first 5 ns (initial) and last 5 ns (final) of the MD simulations for models: (a) F1, (b) F2, and (c) F3.

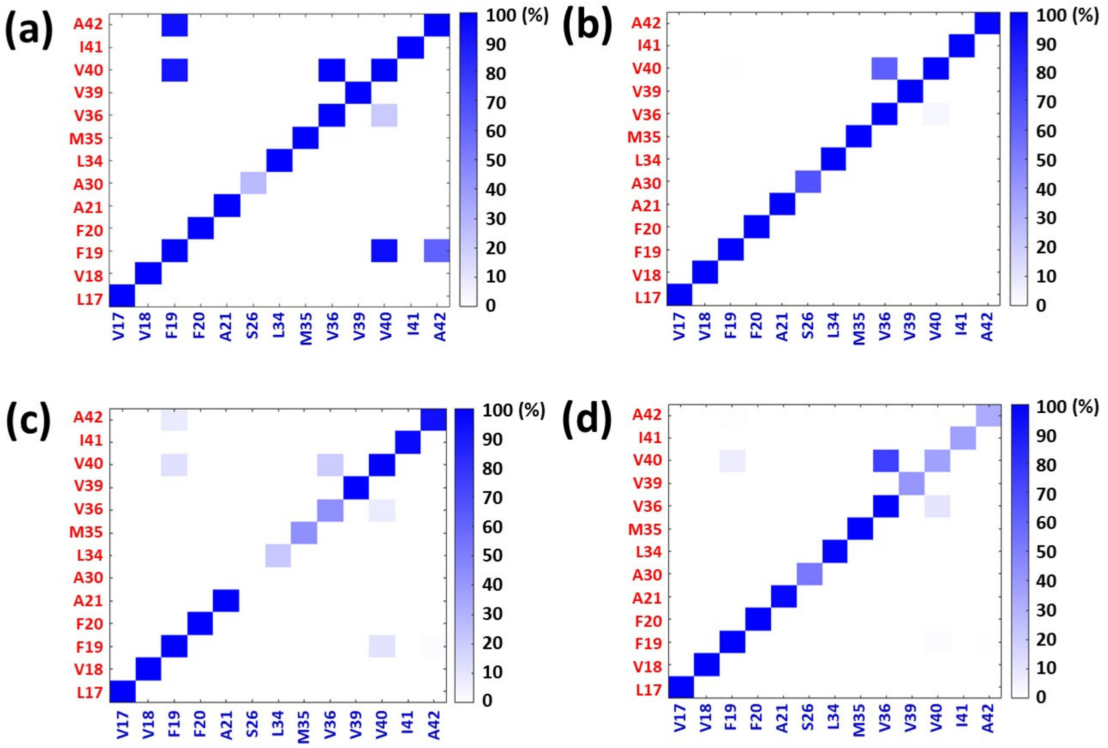

Figure S9: Percentage occurrence maps of hydrophobic interactions between two monomers, computed from MD simulations for (a) Aβ in solution in absence of IDE (taken from: Press-Sandler, O.; Miller, Y., Distinct Primary Nucleation of Polymorphic Aβ Dimers Yields to Distinguished Fibrillation Pathways. *ACS Chem. Neurosci.* 2019, 10, 4407-4413), (b) Aβ in model F1 (c) Aβ in model F2, and (d) Aβ in model F3.

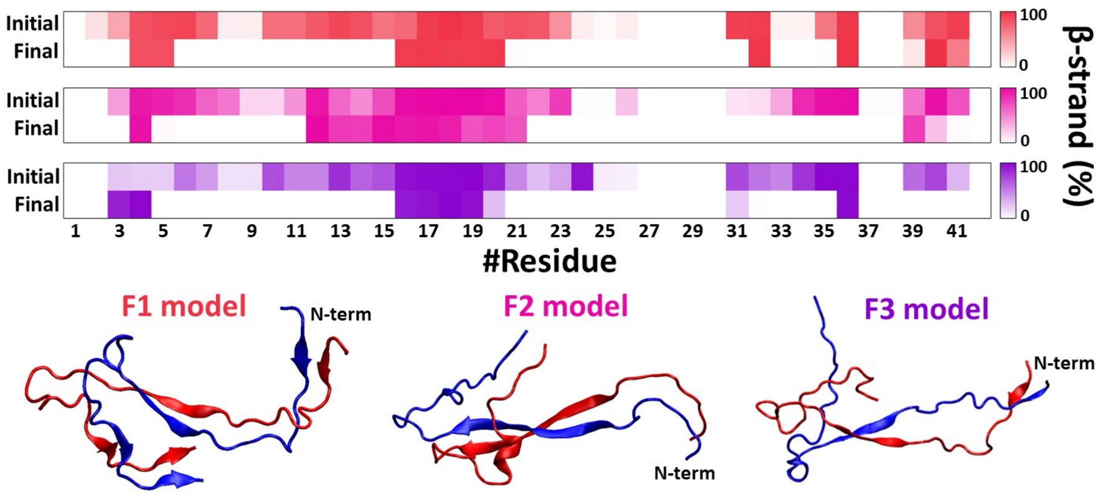

Figure S10: Top: DSSP analysis of the location of β-strands along the sequence of Aβ monomers in the simulated models: F1 (color: red), F2 (color: pink) and F3 (color: purple). The DSSP analyses were measured along the first 5 ns (initial), and the last 5 ns (final) of the MD simulations. Bottom: Final simulated structures of Aβ dimers for each of 3 different models: F1, F2 and F3.

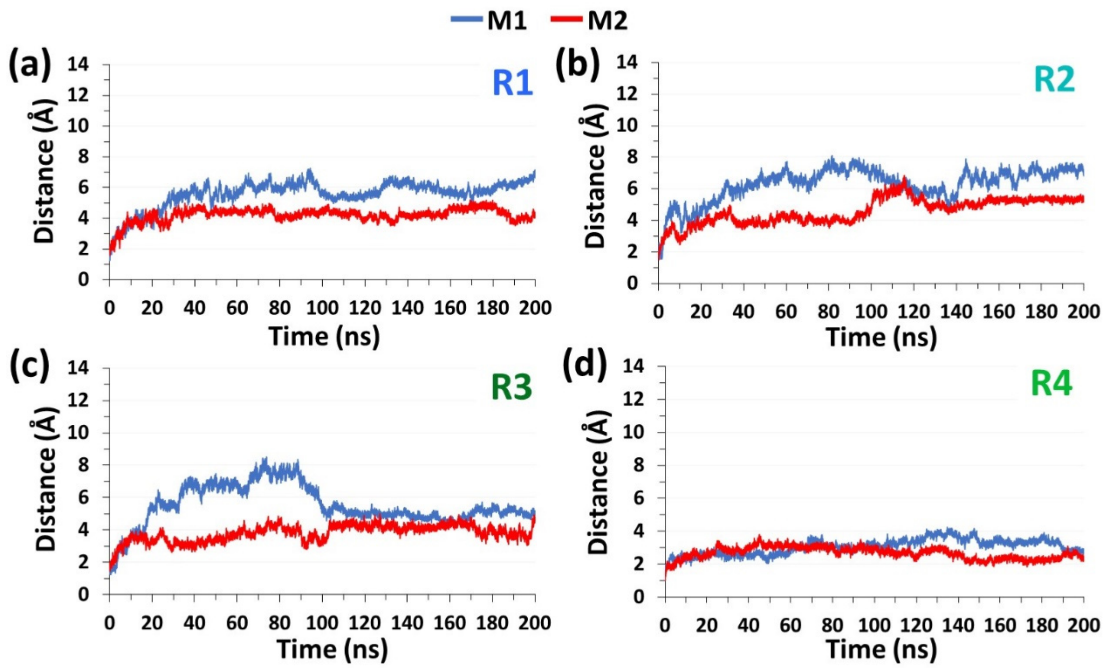

Figure S11: The root-mean-square deviations (RMSDs) values for Aβ dimers in models: (a) R1, (b) R2, (c) R3, and (d) R4, along the MD simulations.

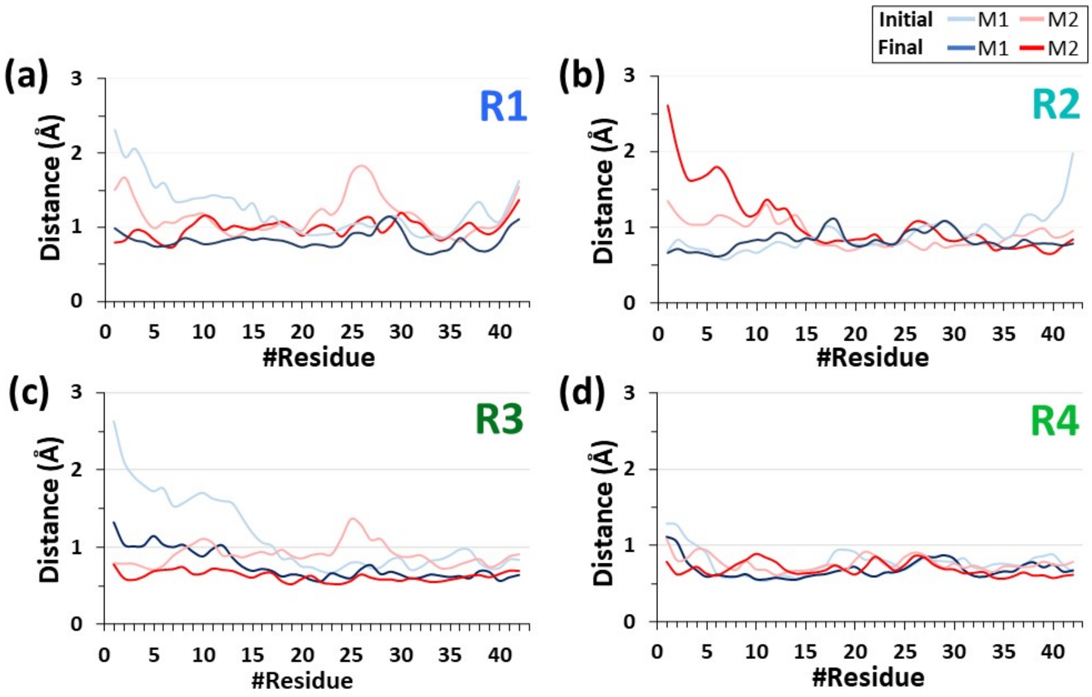

Figure S12: Root-mean-square fluctuations (RMSFs) of Aβ monomers M1 and M2 within each dimer, in the first 5 ns (initial) and last 5 ns (final) of the MD simulations for models: (a) R1, (b) R2, (c) R3, and (d) R4.

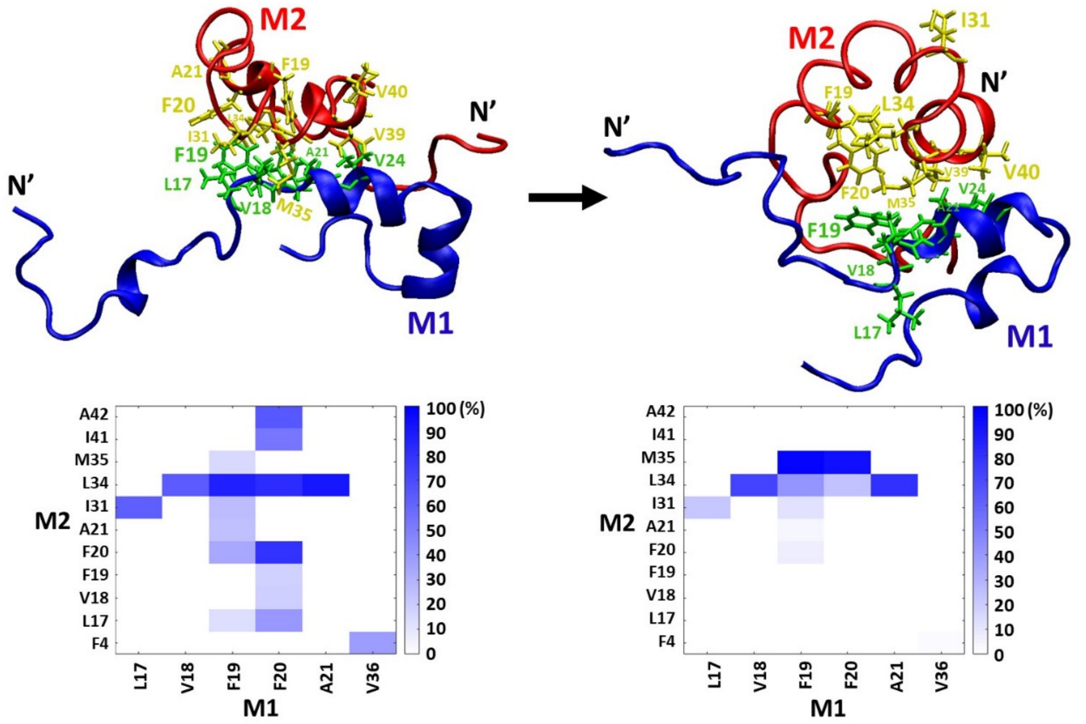

Figure S13: Structure of Aβ dimer – model D1 (top), and percentage occurrence maps of hydrophobic interactions between two monomers, computed from MD simulations for Aβ in solution in absence of IDE (left) (taken from: Press-Sandler, O.; Miller, Y., Distinct Primary Nucleation of Polymorphic Aβ Dimers Yields to Distinguished Fibrillation Pathways. *ACS Chem. Neurosci.* 2019, 10, 4407-4413), and in model R1 (right).

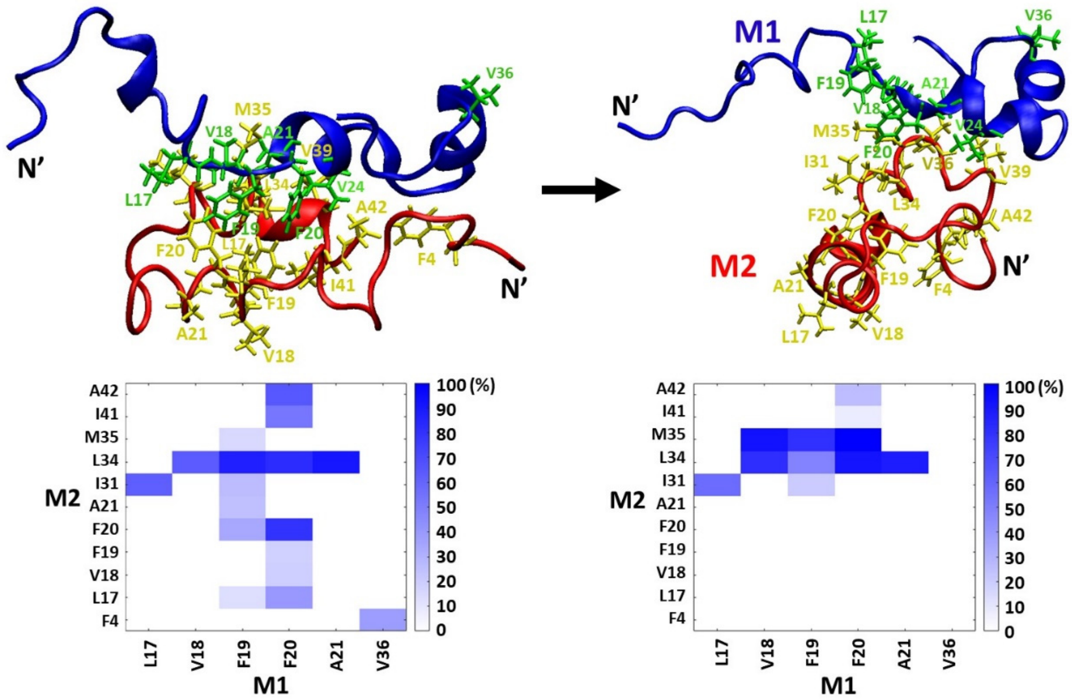

Figure S14: Structure of Aβ dimer – model D1 (top), and percentage occurrence maps of hydrophobic interactions between two monomers, computed from MD simulations for Aβ in solution in absence of IDE (left) (taken from: Press-Sandler, O.; Miller, Y., Distinct Primary Nucleation of Polymorphic Aβ Dimers Yields to Distinguished Fibrillation Pathways. *ACS Chem. Neurosci.* 2019, 10, 4407-4413), and in model R2 (right).

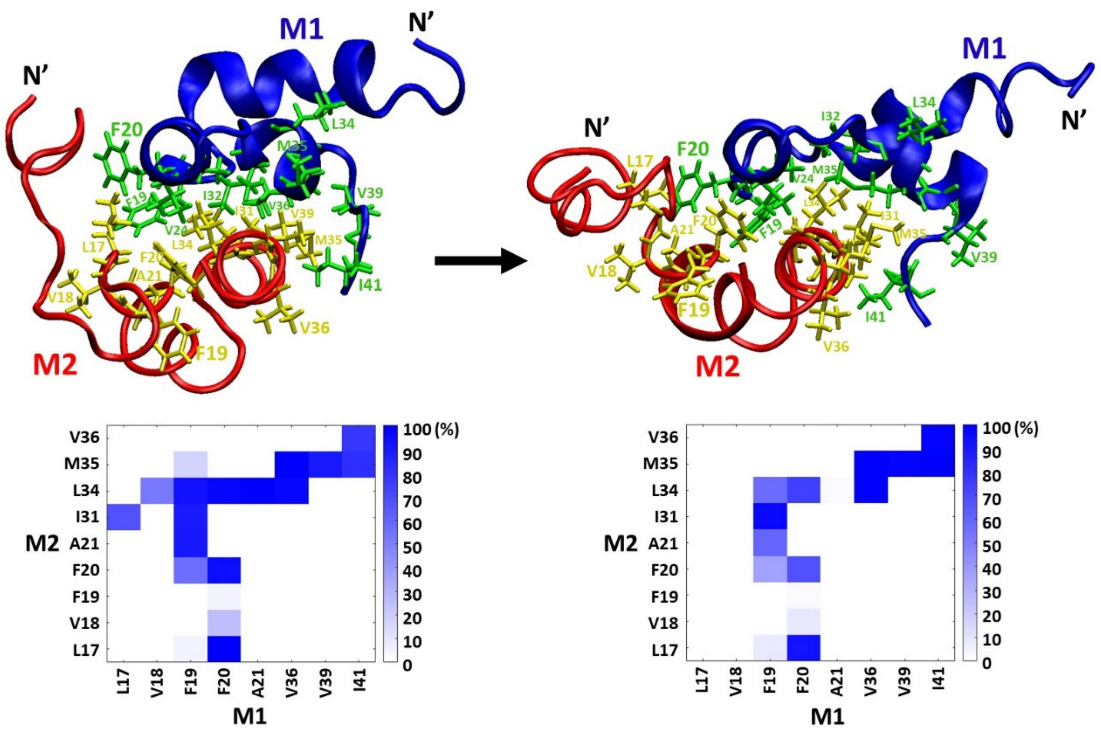

Figure S15: Structure of Aβ dimer – model D4 (top), and percentage occurrence maps of hydrophobic interactions between two monomers, computed from MD simulations for Aβ in solution in absence of IDE (left) (taken from: Press-Sandler, O.; Miller, Y., Distinct Primary Nucleation of Polymorphic Aβ Dimers Yields to Distinguished Fibrillation Pathways. *ACS Chem. Neurosci.* 2019, 10, 4407-4413), and in model R3 (right).

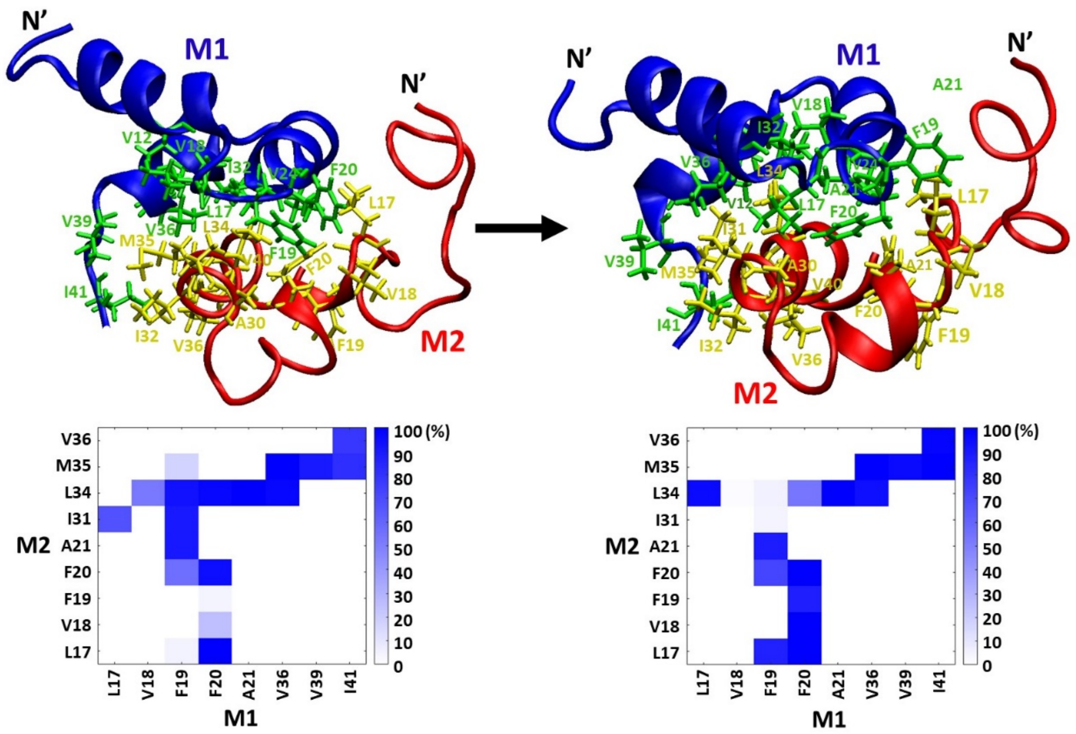

Figure S16: Structure of Aβ dimer – model D4 (top), and percentage occurrence maps of hydrophobic interactions between two monomers, computed from MD simulations for Aβ in solution in absence of IDE (left) (taken from: Press-Sandler, O.; Miller, Y., Distinct Primary Nucleation of Polymorphic Aβ Dimers Yields to Distinguished Fibrillation Pathways. *ACS Chem. Neurosci.* 2019, 10, 4407-4413), and in model R4 (right).

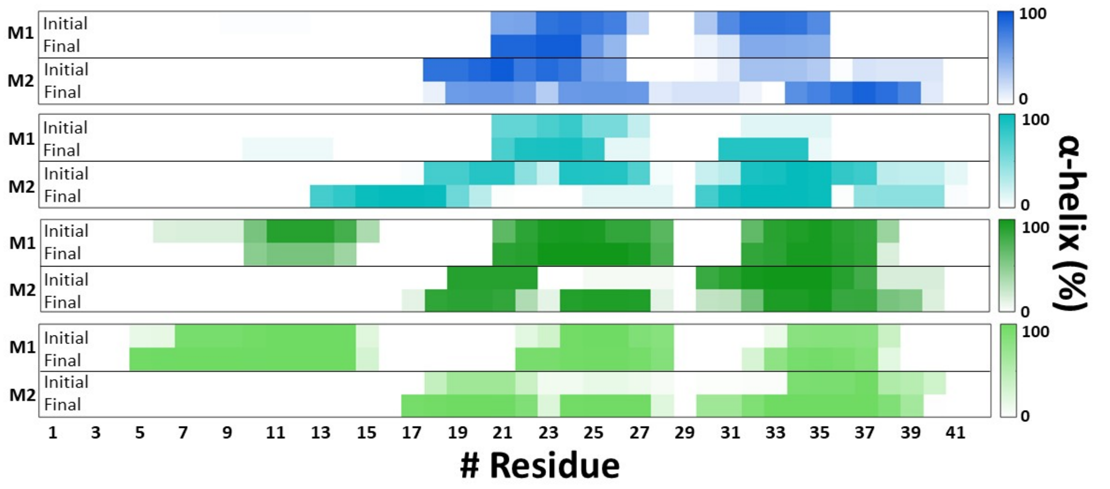

Figure S17: DSSP analysis of the location of α-helices along the sequence of Aβ monomers in the simulated models: R1 (color: blue), R2 (color: cyan) R3 (color: dark green), and R4 (color: light green). The DSSP analyses were measured along the first 5 ns (initial), and the last 5 ns (final) of the MD simulations.

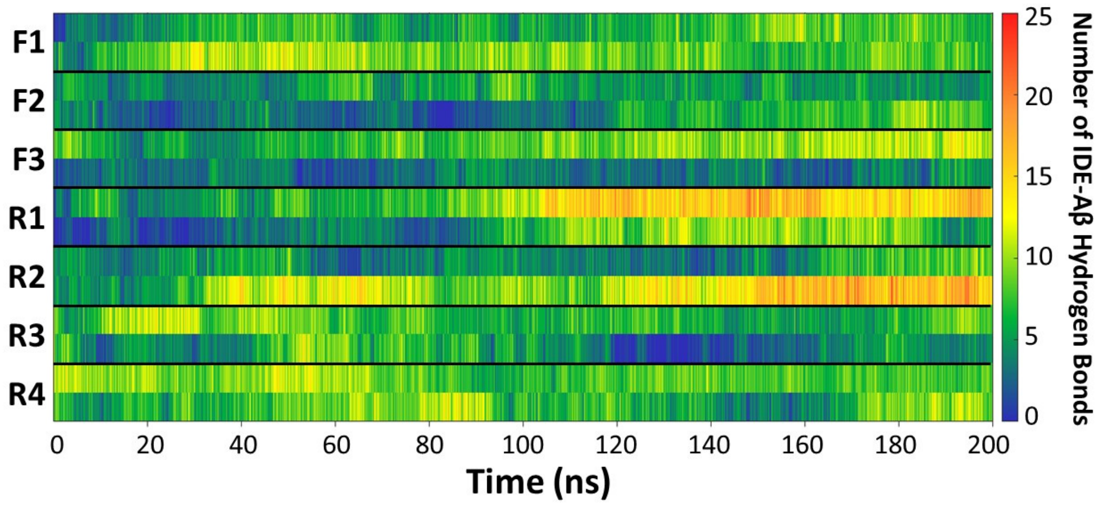

Figure S18: Total number of hydrogen bonds between IDE and Aβ monomers for all models, along the MD simulations.

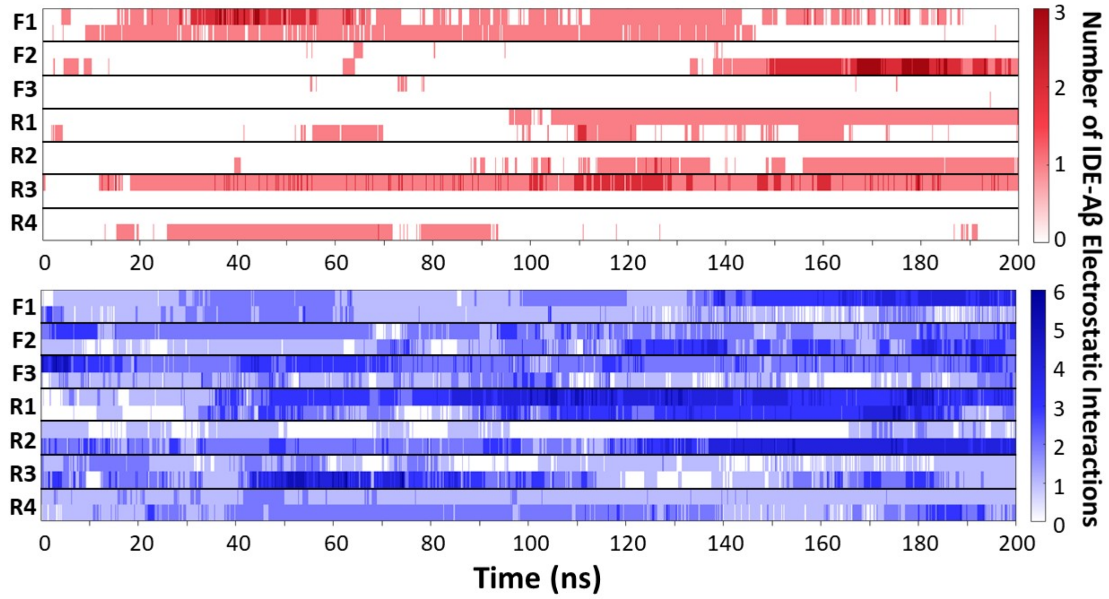

Figure S19: Total number of electrostatic interactions between negative residues (color: red) and positive residues (color: blue) of IDE and Aβ monomers for all models, along the MD simulations.

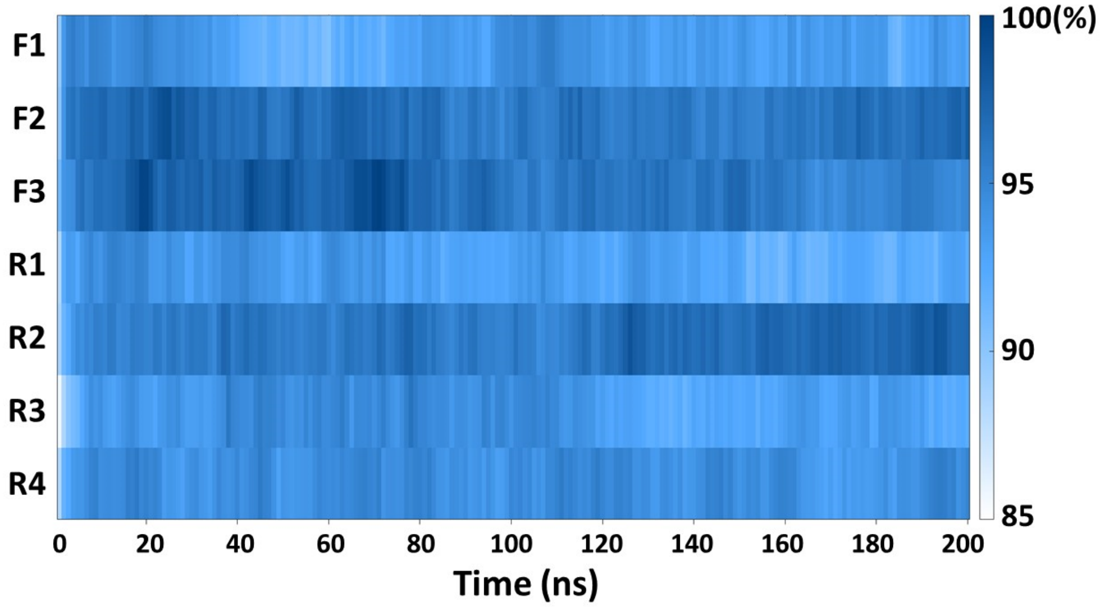

Figure S20: Solvation by water molecules of IDE along the MD simulations for all models. The solvation was normalized to percentages according to the highest number of water molecules.

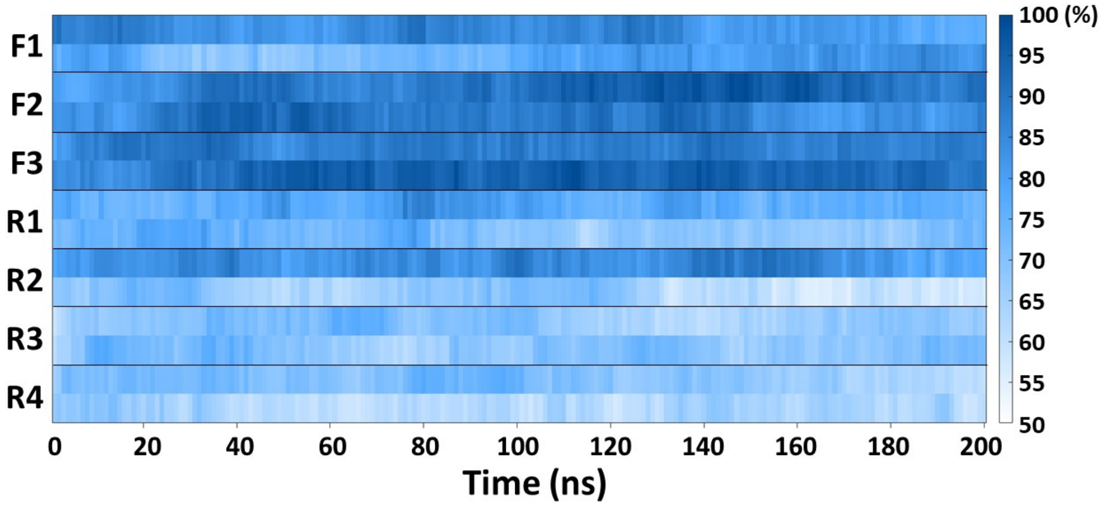

Figure S21: Solvation by water molecules of Aβ monomers along the MD simulations for all models. The solvation was normalized to percentages according to the highest number of water molecules.
